# Dietary flavanols restore hippocampal-dependent memory in older adults with lower diet quality and lower habitual flavanol consumption

**DOI:** 10.1073/pnas.2216932120

**Published:** 2023-05-30

**Authors:** Adam M. Brickman, Lok-Kin Yeung, Daniel M. Alschuler, Javier I. Ottaviani, Gunter G. C. Kuhnle, Richard P. Sloan, Heike Luttmann-Gibson, Trisha Copeland, Hagen Schroeter, Howard D. Sesso, JoAnn E. Manson, Melanie Wall, Scott A. Small

**Affiliations:** ^a^Taub Institute for Research on Alzheimer’s Disease and the Aging Brain, Vagelos College of Physicians and Surgeons, Columbia University, New York, NY 10032; ^b^Gertrude H. Sergievsky Center, Vagelos College of Physicians and Surgeons, Columbia University, New York, NY 10032; ^c^Department of Neurology, Vagelos College of Physicians and Surgeons, Columbia University, New York, NY 10032; ^d^Mental Health Data Science Area, New York State Psychiatric Institute, New York, NY 10032; ^e^Mars Inc., 6885 Elm St, McLean, VA 22101; ^f^Department of Food and Nutritional Sciences, Hugh Sinclair Unit of Human Nutrition, University of Reading, Reading RG6 6DZ, United Kingdom; ^g^Department of Psychiatry, Columbia University Irving Medical Center, New York, NY 10032; ^h^Division of Preventive Medicine, Department of Medicine, Brigham and Women’s Hospital, Harvard Medical School, Boston, MA 02115; ^i^Department of Environmental Health, Harvard T.H. Chan School of Public Health, Boston, MA 02115; ^j^Department of Epidemiology, Harvard T.H. Chan School of Public Health, Boston, MA 02115

**Keywords:** cognitive aging, hippocampus, dietary flavanols

## Abstract

Dietary flavanols are food constituents found in certain fruits and vegetables that have been linked to cognitive aging. Previous studies suggested that consumption of dietary flavanols might specifically be associated with the hippocampal-dependent memory component of cognitive aging and that memory benefits of a flavanol intervention might depend on habitual diet quality. Here, we tested these hypotheses in the context of a large-scale study of 3,562 older adults, who were randomly assigned to a 3-y intervention of cocoa extract (500 mg of cocoa flavanols per day) or a placebo [(COcoa Supplement and Multivitamin Outcomes Study) COSMOS-Web, NCT04582617]. Using the alternative Healthy Eating Index in all participants and a urine-based biomarker of flavanol intake in a subset of participants [n = 1,361], we show that habitual flavanol consumption and diet quality at baseline are positively and selectively correlated with hippocampal-dependent memory. While the prespecified primary end point testing for an intervention-related improvement in memory in all participants after 1 y was not statistically significant, the flavanol intervention restored memory among participants in lower tertiles of habitual diet quality or habitual flavanol consumption. Increases in the flavanol biomarker over the course of the trial were associated with improving memory. Collectively, our results allow dietary flavanols to be considered in the context of a depletion–repletion paradigm and suggest that low flavanol consumption can act as a driver of the hippocampal-dependent component of cognitive aging.

## Significance

Just like there are specific constituents of our diets that are vital for the developing brain, other dietary constituents might be important for the aging brain. This study, a culmination of 15 y of research from mice to humans, provides biomarker-based evidence that dietary consumption of flavanols, a food constituent found in certain fruits and vegetables, can be etiologically linked to age-related memory decline.

“Cognitive aging” is a term used to describe how some of our cognitive abilities decline during the aging process, independent of late-life cognitive diseases (1). Cognitive testing over the life span of humans and other mammals, complemented by neuroimaging and postmortem findings, has established that a key memory component of this profile localizes to the hippocampus (2). Because cognitive aging is meaningfully disruptive to our lives, it is biomedically justified to identify its etiologic factors (3). Identifying a treatable etiology that is linked to lifestyle and diet could have a major public health impact due to its accessibility and relative affordability and safety (4).

Studies suggest that habitual dietary patterns, diet quality, and even specific dietary constituents might represent etiologic factors that affect cognitive aging (5). Dietary flavanols, bioactive compounds commonly found in tea, apples, berries, grapes, cocoa, and other fruits and vegetables, are one such dietary constituent. Dietary intervention studies demonstrated that flavanol consumption can play a role in attenuating cognitive aging by improving hippocampal-dependent memory (6). Flavanols affect angiogenesis (7), and neurovascular indicators suggest that flavanols might improve hippocampal-dependent memory via increased angiogenesis and regional perfusion (6, 8).

In a 3-mo, randomized, placebo-controlled trial (9), we recently investigated the role of a dietary flavanol intervention on memory and cognitive aging across a range of flavanol intake amounts and used measures to evaluate hippocampal-dependent as well as prefrontal-dependent cognition, the two cortical brain areas most sensitive to the aging process and which characterizes cognitive aging. Besides showing that flavanols are differentially linked to hippocampal-dependent cognition, the more important observation was that the most consistent flavanol-induced memory improvement was observed in participants in the lowest tertile of habitual diet quality as assessed by the alternative Healthy Eating Index (aHEI) (10, 11). Because, among others, the index also reflects habitual consumption of foods that are the main sources of dietary flavanols, this observation led to the hypothesis that flavanols might play an etiological role in driving age-related hippocampal dysfunction in older people.

We set out to confirm these observations by integrating multiple longitudinal cognitive assessments into an ongoing large-scale, multiyear, randomized, double-blind placebo-controlled trial, the COcoa Supplement and Multivitamin Outcomes Study (COSMOS; NCT02422745) through an ancillary study COSMOS-Web (NCT04582617) that used web-based cognitive assessments. To address the motivating question, we focused on the one arm of COSMOS in which either a flavanol-containing cocoa extract [500 mg of cocoa flavanols, including 80 mg of (–)-epicatechin] or a placebo was given daily to more than 3,500 generally healthy older men and women. Besides testing whether the flavanol intervention might improve hippocampal-dependent memory, especially in those with relatively poorer habitual diets, the study also allowed us to test the effect over extended durations. Additionally, given its size and scope, COSMOS allowed us to test at scale whether dietary flavanols are indeed differentially linked to hippocampal and not to frontal cortex function. Finally, because many of the COSMOS participants provided urine samples, we were able to apply a validated urine-based biomarker of dietary flavanol consumption (12, 13), a methodological approach critical for understanding the relationship between flavanol consumption and cognition.

In the context of COSMOS-Web, determining whether the flavanol intervention improved hippocampal-dependent memory across all participants at year 1 was the prespecified primary end point, and whether the intervention improved hippocampal-dependent memory primarily in participants with relatively lower habitual diet quality was the prespecified secondary end point.

## Results

### Baseline Characteristics.

**Fig. 1.**
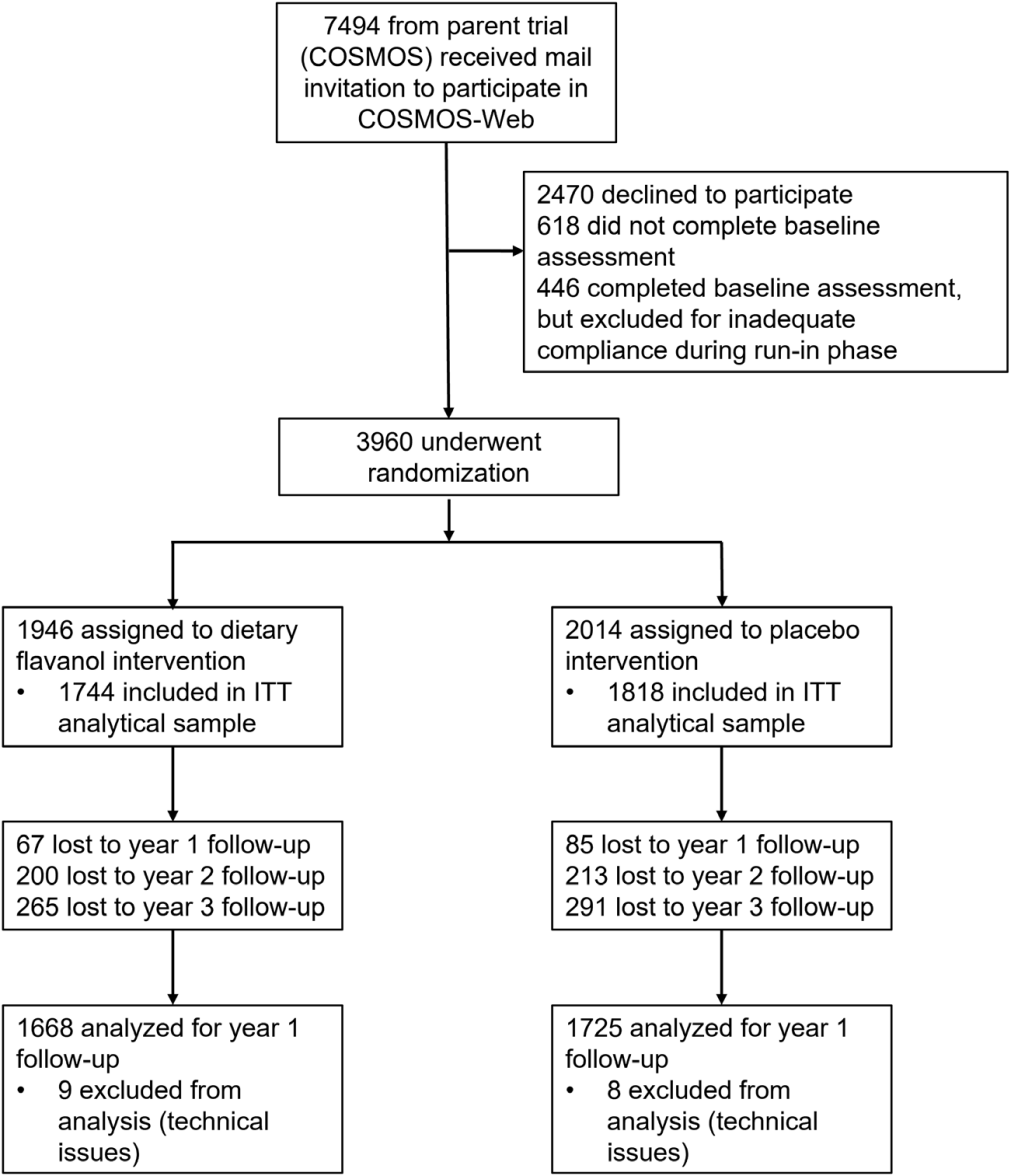
Consolidated Standards of Reporting Trials (CONSORT) diagram for the COSMOS-Web ancillary study.

Characteristics of the 3,562 participants included in the analyses are shown in Table 1 [see the CONSORT (Consolidated Standards of Reporting Trials) diagram, Fig. 1]. Participants were on average 71 y old, predominantly non-Hispanic/non-Latinx White, and the ratio of the number of men to women was 1:2. Participants had high levels of formal education, with the majority reporting college or postcollegiate educational attainment. Importantly, baseline demographic characteristics did not differ between the flavanol intervention and the placebo group, indicating a successful randomization process. Other relevant background and medical characteristics were also similar between treatment conditions in the parent COSMOS trial (14).

**Table 1.** Baseline demographic data, dietary levels, and cognitive test performance by treatment group

	Total Sample (N = 3,562)		Placebo (N = 1,818)		Dietary Flavanol (N = 1,744)		
Measure	N	% or M (SD)	N	% or M (SD)	N	% or M (SD)	Absolute standardized mean difference^*^	*P*-value^†^
Demographic data								
Age	3,562	71.0 (4.6)	1,818	71.0 (4.6)	1,744	71.0 (4.6)	0.003	0.935
Gender								0.790
Men	1,180	33.1%	606	33.3%	574	32.9%	0.009	
Women	2,382	66.9%	1212	66.7%	1170	67.1%	0.009	
Race								0.149
White	3,322	93.3%	1691	93.0%	1631	93.5%	0.020	
African American	88	2.5%	48	2.6%	40	2.3%	0.022	
Hispanic/Latinx	51	1.4%	31	1.7%	20	1.1%	0.047	
Asian or Pacific Islander	54	1.5%	20	1.1%	34	1.9%	0.070	
American Indian or Alaska native	6	0.2%	4	0.2%	2	0.1%	0.026	
Other or unknown	41	1.2%	24	1.3%	17	1.0%	0.032	
Education								0.316
Missing	23	0.6%	12	0.7%	11	0.6%	0.004	
High school	173	4.9%	78	4.3%	95	5.4%	0.054	
College	1,387	38.9%	726	39.9%	661	37.9%	0.042	
Postcollege	1,979	55.6%	1002	55.1%	977	56.0%	0.018	
Baseline dietary measures								
aHEI	3,344	43.3 (10.8)	1705	43.2 (11.0)	1639	43.4 (10.6)	0.018	0.606
Urine gVLM	1,361	9.5 (14.7)	662	9.4 (15.5)	699	9.6 (14.0)	0.008	0.878
Baseline cognitive test scores								
ModRey immediate recall, number of words	3,560	7.16 (3.22)	1817	7.12 (3.24)	1743	7.19 (3.21)	0.021	0.527
ModBent correct rejection, RT (ms)	3,548	2759.56 (1333.59)	1813	2740.06 (1326.21)	1735	2779.93 (1341.34)	0.030	0.373
Flanker direction effect, RT difference (ms)	3,561	31.50 (57.93)	1817	32.17 (58.40)	1744	30.79 (57.45)	0.024	0.477

^*^Standardized absolute mean difference is calculated as the difference between treatment groups divided by the overall SD. Values >0.25 are considered to be nontrivial imbalance due to chance.

^†^*P* values for *t* tests of continuous variables and chi-square tests of categorical variables.

Performance at baseline on the three cognitive measures, the ModRey (primary outcome), Flanker test (secondary outcome), and ModBent (secondary outcome), did not differ between the two intervention groups, and performance metrics were consistent with our previous studies with in-person administration of the cognitive assessment instruments (9) (Table 1). The habitual diet quality of the study cohort, as assessed with the aHEI at study baseline, was 43.5 (median, interquartile range [IQR] = 35.5 to 50.5) and was thus similar-to- moderately better than the habitual diet quality of the average US population (15). Habitual flavanol consumption, as estimated by urinary concentrations of 5-(3′,4′-dihydroxyphenyl)-γ-valerolactone metabolite (gVLM) concentrations at study baseline, had a median (IQR) value of 3.58 µM (0.80 to 10.97 µM) and mean ± SD value of 9.50 ± 14.74 µM, which are very comparable to other large-scale cohorts previously investigated (12). When restricting the sample to those with available gVLM concentrations, baseline demographic, dietary, biomarker, and cognitive characteristics did not differ between randomized groups and were comparable to the overall sample (*SI Appendix*, Table S1).

### Retention and Compliance.

A total of n = 3,960 subjects were randomized and completed the baseline COSMOS-Web assessment, and n = 3,562 (90.0%) participants completed at least one yearly follow-up cognitive assessment and comprise the intention-to-treat (ITT) analytic sample. The proportion of randomized participants who make up the ITT analytic sample was similar between groups [n = 1,744 of 1,946 (89.6%) in the dietary flavanol group; n = 1,818 of 2,014 (90.3%) in the placebo group]. Completion of follow-up measurements by COSMOS participants for the primary outcome, ModRey, at years 1, 2, and 3 among the ITT analytic sample was 95.2%, 87.3%, and 82.9%, respectively.

Pill compliance (not missing >8 d/mo of study pills) based on self-reported pill counts was high among the ITT sample at each year (years 1, 2, and 3: 91.3%, 88%, and 86.7%, respectively) with no significant differences between randomized groups at year 1 (*P* = 0.942) and year 2 (*P* = 0.634), but a significant difference between groups at year 3 (*P* = 0.047). Participants in the ITT sample who also met pill compliance were included in the per-protocol (PP) analyses.

### The Flavanol Intervention Did Not Enhance Memory Among All Participants.

As in the previous trial (9), the ModRey was used as the cognitive measure of hippocampal memory and was prespecified as the primary outcome. The Color/Directional Flanker Task, an established cognitive measure of prefrontal cortex function (16), was prespecified as a secondary end point and was predicted to remain unaffected by the intervention. The utility of the ModBent, a recently developed measure designed to be sensitive to dentate gyrus function, was tested in the previous trial and found to be psychometrically suboptimal, particularly when applied to older people, as it yielded near-chance performance in post hoc analyses (9). Nevertheless, it was also prespecified as a secondary end point in this trial.

In our primary analysis, when examining performance on the ModRey at year 1, participants in both the flavanol and placebo groups showed a typical learning (practice) effect, but no treatment differences were observed, with similar magnitude of improvements observed in the two groups (*d* = 0.025, *P* = 0.42, Table 2). In a separate analysis performed in the PP sample (*SI Appendix*, Table S2), no treatment difference on the ModRey was found.

**Table 2.** Results of the longitudinal mixed-effect models examining primary (ModRey at year 1) and secondary (ModBent and Flanker) outcomes by randomized assignment

	Placebo				Dietary flavanol				Treatment Difference		
Time point	N	Raw mean (SD)	Raw mean change from baseline (SD)	Within group *P*-value	N	Raw mean (SD)	Raw Mean change from baseline (SD)	Within group *P*-value	Mean (SE)^*^	Cohen’s d	*P*-value
ModRey: Immediate recall, number of words											
Baseline	1,817	7.12 (3.24)			1,743	7.19 (3.21)					
Year 1^†^	1,725	7.67 (3.38)	0.53 (3.32)	<0.001	1,668	7.79 (3.27)	0.60 (3.31)	<0.001	0.08 (0.10)	0.025	0.415
Year 2	1,583	7.99 (3.38)	0.83 (3.40)	<0.001	1,525	8.10 (3.26)	0.85 (3.43)	<0.001	0.04 (0.10)	0.013	0.685
Year 3	1,496	8.22 (3.41)	1.07 (3.29)	<0.001	1,456	8.22 (3.41)	0.98 (3.32)	<0.001	-0.06 (0.11)	0.018	0.591
ModBent: Correct rejection, RT (ms)											
Baseline	1,778	2,740.66 (1,329.47)			1,709	2,781.60 (1,342.96)					
Year 1	1,652	2,762.12 (1,276.26)	24.74 (1,500.55)	0.740	1,592	2,844.06 (1,450.55)	65.78 (1,674.51)	0.014	68.76 (45.16)	0.051	0.128
Year 2	1,523	2,774.03 (1,369.69)	29.30 (1,561.36)	0.525	1,468	2,801.02 (1,344.27)	25.10 (1,603.54)	0.281	15.20 (46.85)	0.011	0.746
Year 3	1,438	2,816.45 (1,306.71)	76.83 (1,487.01)	0.082	1,392	2,799.84 (1,411.45)	47.20 (1,579.54)	0.236	-17.97 (48.00)	0.013	0.708
Flanker: Direction effect, RT difference (ms)											
Baseline	1,780	31.70 (57.66)			1,715	30.41 (57.16)					
Year 1	1,655	26.22 (57.49)	−5.53 (74.83)	<0.001	1,597	27.69 (50.62)	−2.61 (70.06)	0.011	1.69 (1.86)	0.029	0.363
Year 2	1,519	26.33 (50.02)	−5.15 (70.59)	<0.001	1,473	27.37 (50.94)	−3.82 (68.82)	0.007	1.24 (1.93)	0.022	0.523
Year 3	1,434	23.51 (56.27)	−8.05 (77.33)	<0.001	1,402	24.11 (54.64)	−7.01 (73.36)	<0.001	0.62 (1.99)	0.011	0.754

^*^Treatment effect controlling for baseline.

^†^Prespecified primary outcome

Similarly, the flavanol intervention did not affect the prespecified secondary cognitive outcome, ModBent at year 1 (d = 0.051 at year 1, *P* = 0.13). As hypothesized, it did not affect performance on the Flanker test (d = 0.029 at year 1, *P* = 0.36). No statistically significant differences for all three cognitive measures were found at year 2 or 3.

### The Flavanol Intervention Improved Memory in Participants in the Lowest Tertile of Habitual Diet Quality.

Based on our previous study (9), a key hypothesis was that the impact of the flavanol intervention on the ModRey would be more pronounced among those with poorer baseline diet quality. To test this hypothesis, we stratified the participants into tertiles based on the overall distribution of baseline aHEI scores (low tertile ≤ 38; medium tertile 38 to 48; high tertile ≥ 48) in the COSMOS-Web subcohort. Compared with the National Health and Nutrition Survey (13), the low aHEI tertile reflects a diet quality ranging from the US average to slightly below average; the medium aHEI tertile ranges from average to well-above average; and the high aHEI tertile includes people with a substantially better diet than that of the average American (15). Consistent with our previous observations (9), those in the lowest tertile of diet quality had poorer memory performance at baseline specific to the hippocampus-mediated ModRey test, but this relationship was not observed in the prefrontal cortex-sensitive Flanker test. These findings remained whether considering diet quality as a continuous measure or divided into tertiles (Table 3).

**Table 3.** Association of the baseline aHEI and gVLM with baseline performance on cognitive outcomes.

	aHEI/gVLM characteristics		ModRey: Immediate recall				ModBent: Correct rejection				Flanker direction effect			
	N	Median (IQR)	N	r or mean (SD)	*P*-value	Adjusted *P*-value^*^	N	r or mean (SD)	*P*-value	Adjusted *P*-value^*^	N	r or mean (SD)	*P*-value	Adjusted *P*-value^*^
aHEI														
Continuous/overall	3,344	43.50 (35.50, 50.50)	3,342	r = 0.101	<0.001	<0.001	3,277	r = 0.034	0.051	0.057	3,286	r = 0.009	0.594	0.737
Low	1,111	32.50 (28.50, 35.50)	1,110	6.75 (3.07)	<0.001	0.005	1,086	2,710.15 (1205.13)	0.257	0.310	1,092	29.03 (56.51)	0.266	0.271
Medium	1,118	43.50 (40.50, 45.50)	1,118	7.27 (3.33)			1,101	2,761.08 (1391.73)			1,098	32.76 (54.37)		
High	1,115	54.50 (50.50, 58.50)	1,114	7.43 (3.25)			1,090	2,802.33 (1312.93)			1,096	30.00 (55.82)		
gVLM														
Continuous/overall	1,361	3.58 (0.80, 10.97)	1,361	r = 0.050	0.066	0.076	1,334	r = 0.027	0.325	0.294	1,333	r = −0.010	0.714	0.852
Low	453	0.42 (0.11, 0.80)	453	6.89 (3.09)	0.065	0.059	441	2,765.71 (1,371.40)	0.961	0.926	441	31.57 (56.46)	0.752	0.651
Medium	454	3.58 (2.29, 5.19)	454	7.01 (3.27)			448	2,739.37 (1,303.52)			447	33.11 (56.26)		
High	454	16.73 (10.97, 30.87)	454	7.37 (3.32)			445	2,758.15 (1,601.50)			445	34.38 (53.98)		

^*^Adjusted for sex, age, education, and race.

**Fig. 2.**
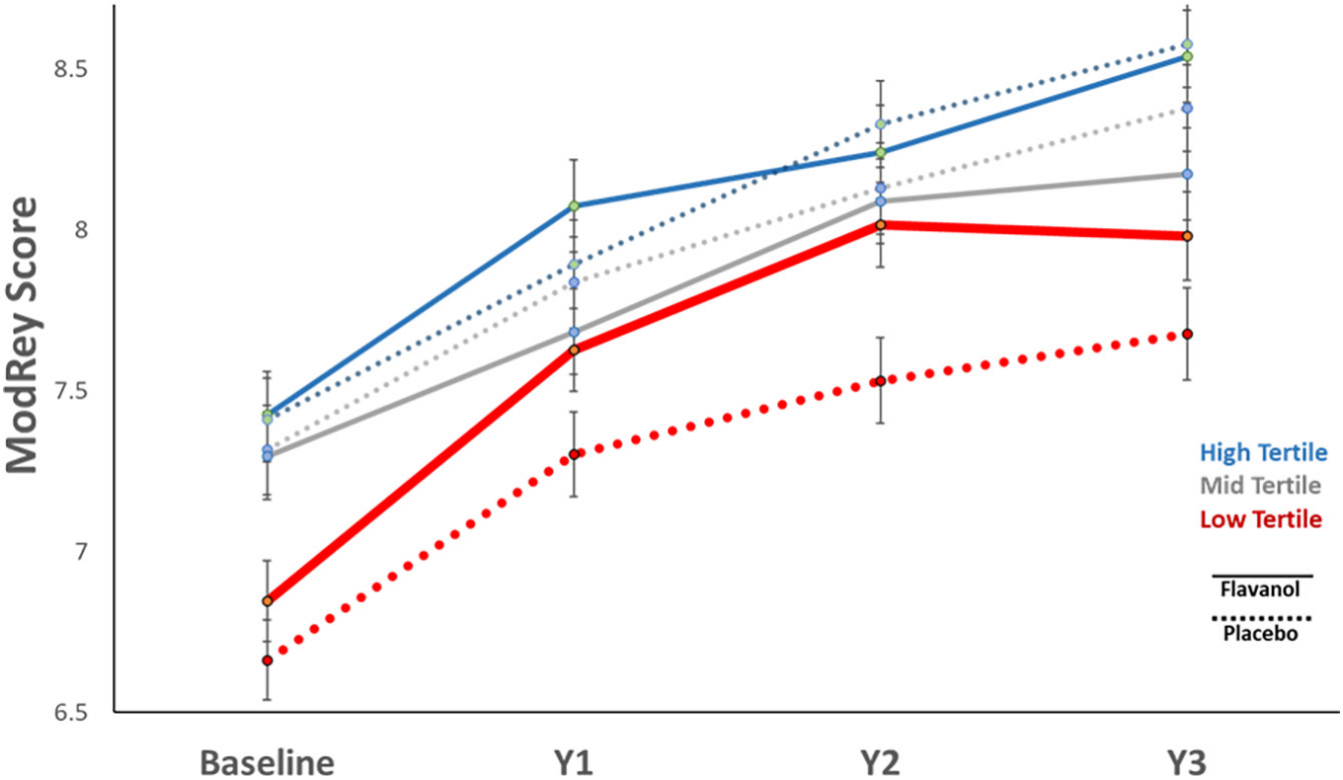
Improvement in ModRey performance by baseline aHEI.

As hypothesized, the flavanol effects on ModRey were statistically different across aHEI groups (interaction test F = 3.21, d = 2, *P* = 0.041). In particular, the flavanol intervention improved performance on the ModRey test compared to placebo in participants in the low aHEI tertile (overall effect: d = 0.086, p = 0.011) but not among those with a medium or high aHEI at baseline (Table 4). Fig. 2 shows the greater improvement for those on flavanol compared to placebo in the low aHEI group.

**Table 4.** Planned subgroup analyses: Results of the longitudinal mixed-effect model examining ModRey by flavanol assignment and aHEI tertile^*^

	Placebo				Dietary flavanol				Treatment Difference		
Time point	N	Raw mean (SD)	Raw mean change from baseline (SD)	Within-group *P*-Value	N	Raw mean (SD)	Raw Mean change from baseline (SD)	Within-group *P*-value	Mean difference (SE)^*^	Cohen’s d	*P*-value
Low aHEI tertile											
Baseline	582	6.68 (3.12)			528	6.84 (3.01)					
Year 1	559	7.25 (3.22)	0.57 (3.15)	0.009	506	7.61 (3.01)	0.78 (3.10)	<0.001	0.28 (0.18)	0.088	0.115
Year 2	501	7.52 (3.32)	0.84 (3.26)	<0.001	460	7.96 (3.08)	1.07 (3.16)	<0.001	0.33 (0.19)	0.103	0.078
Year 3	465	7.63 (3.55)	0.90 (3.36)	<0.001	447	7.94 (3.25)	1.02 (3.28)	<0.001	0.21 (0.19)	0.067	0.267
Overall^†^									0.28 (0.11)	0.086	0.011
Medium aHEI tertile											
Baseline	552	7.29 (3.33)			566	7.24 (3.32)					
Year 1	526	7.86 (3.41)	0.60 (3.55)	<0.001	539	7.73 (3.23)	0.53 (3.36)	<0.001	-0.10 (0.18)	-0.032	0.569
Year 2	486	8.09 (3.40)	0.79 (3.62)	<0.001	496	8.10 (3.20)	0.80 (3.56)	<0.001	0.01 (0.19)	0.004	0.945
Year 3	458	8.37 (3.27)	1.08 (3.37)	<0.001	462	8.13 (3.52)	0.87 (3.43)	<0.001	-0.23 (0.19)	-0.071	0.234
Overall^†^									-0.11 (0.11)	-0.033	0.323
High aHEI tertile											
Baseline	570	7.46 (3.23)			544	7.40 (3.28)					
Year 1	533	7.87 (3.34)	0.34 (3.28)	<0.001	520	8.08 (3.43)	0.65 (3.33)	<0.001	0.26 (0.18)	0.082	0.144
Year 2	505	8.34 (3.29)	0.84 (3.42)	<0.001	481	8.21 (3.48)	0.76 (3.47)	<0.001	-0.10 (0.19)	-0.031	0.595
Year 3	488	8.61 (3.31)	1.15 (3.25)	<0.001	460	8.53 (3.41)	1.13 (3.28)	<0.001	-0.05 (0.19)	-0.015	0.806
Overall^†^									0.04 (0.11)	0.012	0.713
Interaction aHEI tertile*flavanol									F = 3.21, df = 2, *P* = 0.041		

^*^Treatment effect controlling for baseline derived from the longitudinal mixed-effects model.

^†^Overall is the contrast estimate within each tertile of the flavanol versus placebo effect averaged across all 3 y derived from the longitudinal mixed-effects model.

### The Flavanol Intervention Improved Memory in Participants in the Lowest Tertile of a Biomarker of Flavanol Consumption.

Our prespecified hypothesis was that hippocampal-dependent memory, as assessed by the ModRey, would improve with dietary flavanol intervention particularly among individuals who have lower aHEI scores at baseline, a measure that is also likely to correlate with lower habitual flavanol consumption. After the commencement of COSMOS, a urine-based biomarker of flavanol intake was developed and validated (12, 13), which allowed us to more directly test whether hippocampal-dependent memory is correlated with habitual flavanol consumption in analyses that were not prespecified but relevant in the context of our findings for the aHEI. Thus, we measured urinary gVLM levels in all COSMOS and COSMOS-Web participants who provided a urine sample (n = 1,361) and stratified this subcohort into tertiles based on urinary gVLM levels, as a measure of habitual flavanol consumption (low tertile < 1.43 µM; medium tertile 1.43 to 7.49 µM; high tertile ≥ 7.50 µM). *SI Appendix*, Table S3 displays the overlap between tertiles defined by baseline gVLM levels and tertiles defined by baseline aHEI levels.

**Fig. 3.**
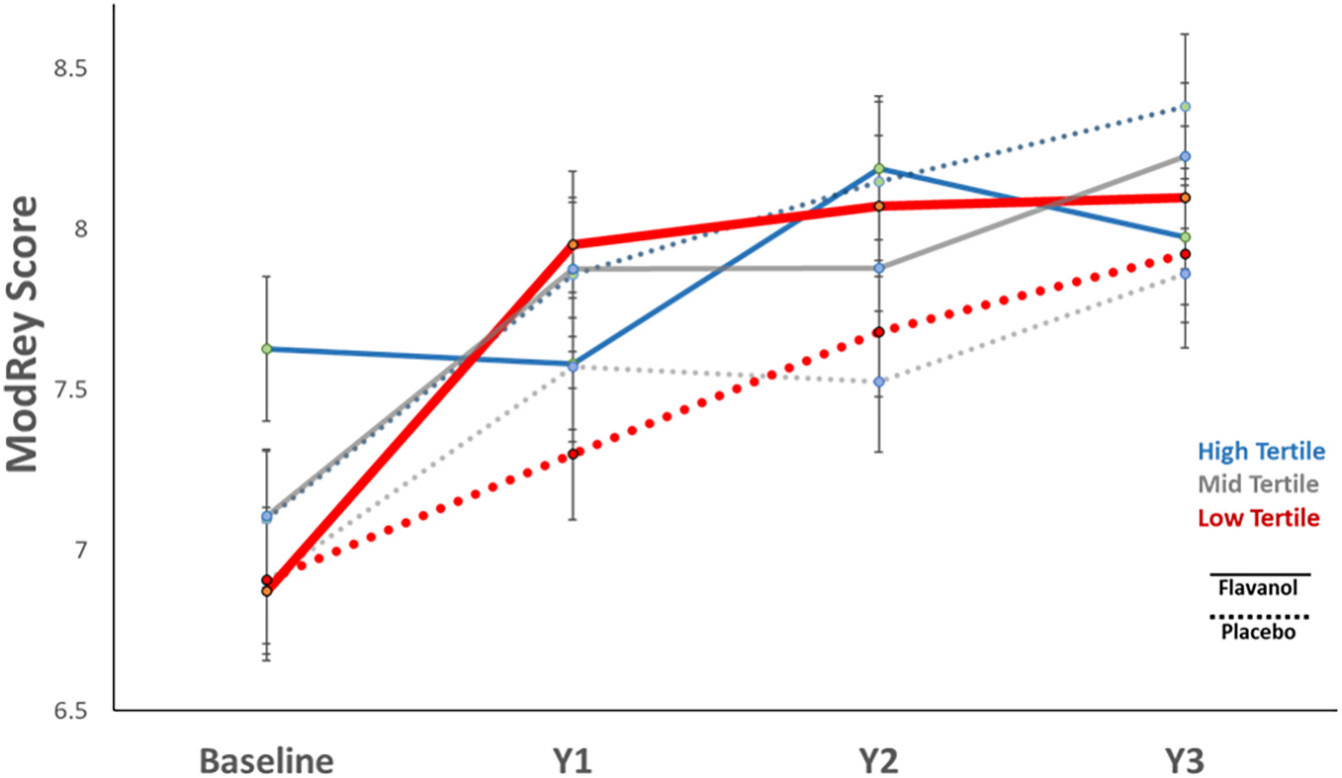
Improvement in ModRey performance by baseline gVLM.

As hypothesized, the flavanol effects on the ModRey were statistically different across gVLM groups (interaction test F = 10.1, d = 2, *P* < 0.001). Our data demonstrate that performance on the ModRey was significantly improved with the dietary flavanol intervention (overall effect: d = 0.141, *P* = 0.006) in the lowest gVLM tertile (Table 5 and Fig. 3). *SI Appendix*, Fig. S1 illustrates the continuous associations between baseline gVLM values and change in ModRey scores.

**Table 5.** Results of the longitudinal mixed-effect model examining ModRey by flavanol assignment and gVLM tertile

	Placebo				Dietary flavanol				Treatment difference		
Time point	N	Raw mean (SD)	Raw mean change from baseline (SD)	Within-group *P*-value	N	Raw mean (SD)	Raw mean change from baseline (SD)	Within-group *P*-value	Mean difference (SE)^*^	Cohen's d	*P*-value
Low gVLM tertile											
Baseline	235	6.91 (3.02)			218	6.87 (3.17)					
Year 1	220	7.30 (3.14)	0.35 (3.33)	0.166	207	7.95 (3.38)	1.16 (3.27)	<0.001	0.73 (0.28)	0.227	0.009
Year 2	213	7.68 (3.12)	0.74 (3.00)	<0.001	195	8.07 (3.24)	1.22 (3.26)	<0.001	0.44 (0.28)	0.135	0.126
Year 3	208	7.92 (3.27)	0.96 (3.41)	<0.001	184	8.10 (3.28)	1.17 (3.26)	<0.001	0.20 (0.29)	0.061	0.501
Overall^†^									0.46 (0.16)	0.141	0.006
Medium gVLM tertile											
Baseline	209	6.90 (3.29)			245	7.11 (3.25)					
Year 1	198	7.57 (3.36)	0.64 (3.45)	0.007	232	7.88 (3.28)	0.75 (3.31)	<0.001	0.21 (0.28)	0.064	0.456
Year 2	181	7.52 (3.16)	0.52 (3.00)	0.028	213	7.88 (3.01)	0.84 (3.28)	<0.001	0.34 (0.29)	0.105	0.246
Year 3	152	7.86 (3.35)	0.95 (3.15)	<0.001	206	8.23 (3.55)	1.10 (3.47)	<0.001	0.26 (0.31)	0.080	0.402
Overall^†^									0.27 (0.17_	0.083	0.114
High gVLM tertile											
Baseline	218	7.10 (3.15)			236	7.63 (3.46)					
Year 1	204	7.86 (3.55)	0.77 (3.01)	<0.001	226	7.58 (3.15)	−0.07 (3.25)	0.337	−0.58 (0.28)	−0.180	0.037
Year 2	188	8.15 (3.65)	1.06 (3.25)	<0.001	217	8.19 (3.44)	0.45 (3.75)	<0.001	−0.30 (0.29)	−0.094	0.289
Year 3	178	8.38 (3.31)	1.40 (2.91)	<0.001	210	7.98 (3.27)	0.39 (3.41)	0.002	−0.73 (0.29)	−0.225	0.013
Overall^†^									−0.54 (0.17)	−0.166	0.001
Interaction gVLM tertile*flavanol									F = 10.1, df = 2, *P*-value < 0.001		

^*^Treatment effect controlling for baseline derived from the longitudinal mixed-effects model.

^†^Overall is the contrast estimate within each tertile of the flavanol versus placebo effect averaged across all 3 y derived from the longitudinal mixed-effects model.

**Fig. 4.**
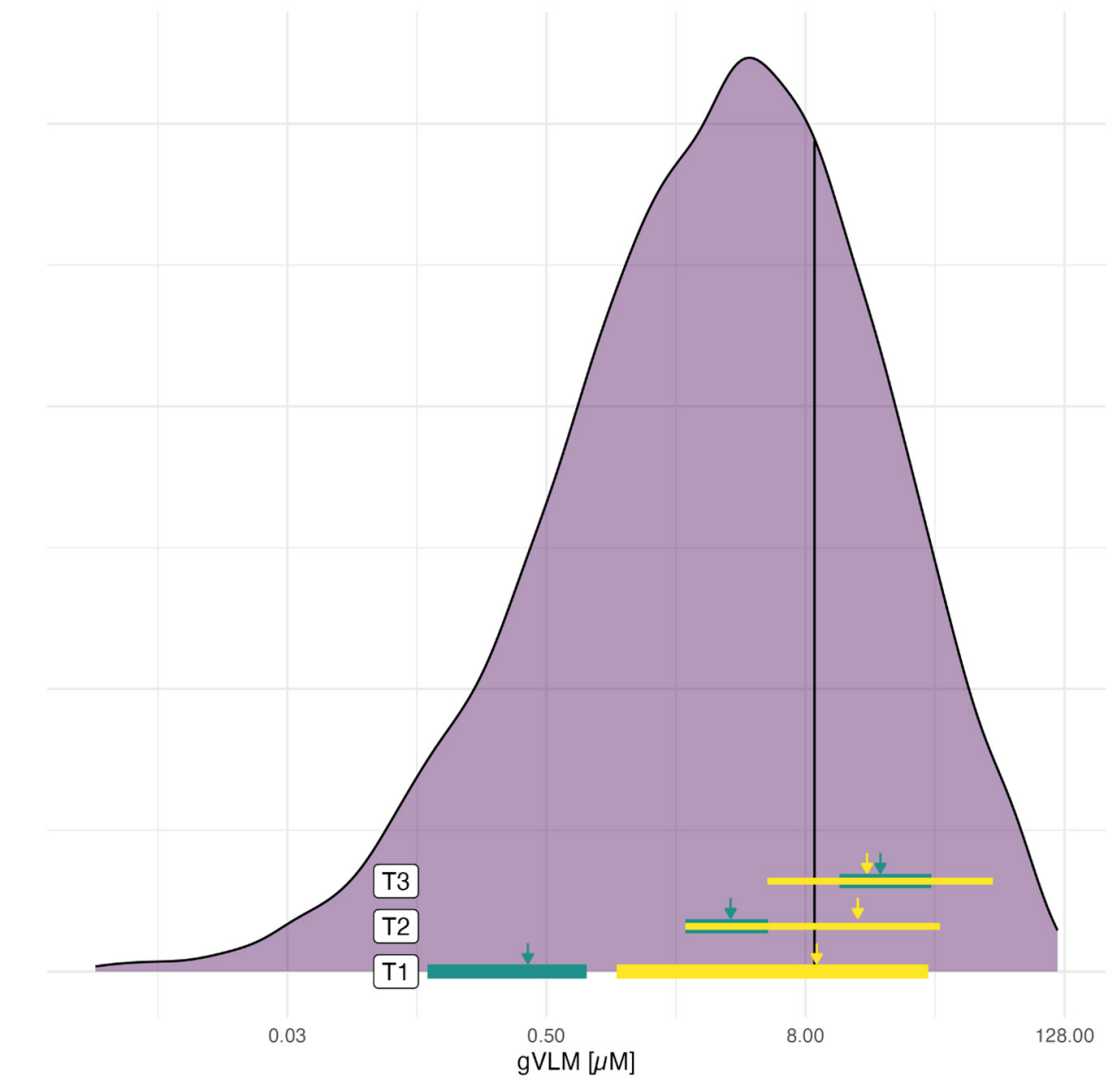
Population distribution of urine gVLM. The mean (arrow) and distribution (colored bar) among those randomized to the flavanol intervention group by gVLM tertile (T1, T2, and T3) are plotted before (in green) and after (in yellow) 1 y of intervention. The data are mapped onto the log distribution of urine gVLM (in purple) with mean value indicated by solid line among 6,003 individuals aged 60 y and older (13).

Further supporting that the memory benefit is related to the flavanol intervention, in a smaller subset who had urine gVLM measures at baseline and again at 1-y follow-up (n = 352), the degree of improvement on the ModRey was associated with the magnitude of increase in gVLM urine concentrations adjusting for treatment group, age, sex, and education (adjusted beta = 0.02, SE = 0.01, *P* = 0.009; *SI Appendix*, Fig. S2 displays the relationship between change in gVLM levels and change in ModRey immediate memory scores). This observation suggests a potential codependency between change in gVLM urine levels and change in memory performance. To further explore this relationship in the context of expected gVLM levels in a reference population, we derived the distribution of gVLM levels from over 6,000 people, 60 y and older, who were part of a prior large-scale epidemiological study (12). As seen in Fig. 4, the mean gVLM levels of COSMOS-Web participants in the lowest tertile that were randomized to the flavanol intervention group were notably below the mean value of gVLM levels in the reference population. After 1 y of the flavanol-based intervention, the mean gVLM urine levels in the low gVLM tertile normalized, as values now matched the mean gVLM levels in the reference population.

Finally, as with the aHEI, there was a similar, though more modest, association between baseline urine gVLM concentrations and ModRey performance when considering gVLM levels as continuous (r = 0.050, *P* = 0.076) or based on tertiles (*P* = 0.059); here, the high tertile group differed significantly from the low tertile in post hoc contrasts (*P* = 0.019) (Table 3). These results replicate our earlier findings of a relationship between diet quality and memory performance (9). More importantly, they introduce observations of an association between gVLM and cognition that is specific to hippocampal-dependent memory function, establishing a codependency between dietary flavanol levels increased via flavanol supplementation and cognition in older adults.

### Safety of the Dietary Flavanol Intervention.

Cocoa-extracted dietary flavanols were generally safe and well tolerated in the main trial (14). No significant effects on a range of nonmonitored cardiovascular, cancer, and other outcomes were observed (all *P* > 0.05) (14). Among self-reported side effects associated with flavanol supplementation, those taking the active supplement were slightly more likely to have nausea and stomach discomfort, but they were slightly less likely to have flu-like symptoms and headaches compared with placebo (14). Dropout and death rates did not differ by treatment condition (14).

## Discussion

In the context of a clinical trial notable for its large scale and long duration, we find that although the prespecified primary end point testing for an intervention-related improvement in memory in all participants after 1 y was not statistically significant, a flavanol intervention-based restoration of memory was observed in the lower tertile of habitual diet quality and in the subset of participants with lower habitual flavanol consumption. The improvement in memory was apparent after 12 mo of intervention and appeared to be sustained over the 3 y of follow-up. Replicating and extending our previous finding in a much larger number of older people and for up to 3 y of follow up, we also found that diet quality is a key lifestyle factor linked to the hippocampal and not to the prefrontal component of cognitive aging, as indexed by performance on neuropsychological instruments that assess these respective systems. We demonstrate that habitual flavanol consumption and diet quality at baseline are positively and selectively correlated with hippocampal-dependent memory. Improvements in the flavanol biomarker over the course of the trial were associated with improving memory.

The experimental findings implicate dietary flavanols as a specific constituent of a healthy diet that when habitual consumption is lower can underlie the hippocampal-dependent memory component of cognitive aging. This interpretation was confirmed in a subset of participants by measuring their urinary gVLM concentrations, a validated biomarker of flavanol consumption. Like diet quality, baseline gVLM levels selectively correlated with hippocampal-dependent memory, and the group with relatively lower gVLM levels exhibited worse baseline memory. In this subset of participants, the observed memory restorative pattern was even more pronounced, and based on a population distribution, their below-average gVLM concentrations effectively normalized after 1 y of the flavanol-based intervention. Furthermore, when investigating the whole sample, an increase in gVLM concentrations over the course of the trial was associated with improving hippocampal-dependent memory. Taken together, the findings allow the causal logic of “depletion–repletion” to be invoked in support of an etiological conclusion: that relatively lower flavanol consumption can act as a dietary driver of the hippocampal-dependent component of cognitive aging.

While we have systematically repleted dietary flavanols in those with habitually lower flavanol consumption at baseline, we did not do the opposite. Therefore, we cannot definitively conclude whether lower dietary intake alone can induce the relatively poorer memory performance observed at baseline. However, given the association between flavanol consumption and memory observed at baseline in this and previous studies, a flavanol depletion experiment in humans, while potentially informative, might be deemed unethical. An alternative approach is for a future trial to enroll participants on the lowest end of the gVLM distribution and to test the prediction that a more dramatic improvement in memory will be observed upon flavanol repletion.

Another reason supporting an etiological conclusion is related to anatomical biology. Showing that a healthy diet in general and flavanols in particular are most reliably linked to the hippocampal and not to the prefrontal component of cognitive aging implies mechanistic specificity. Previous studies in mice demonstrated that dietary flavanols can enhance hippocampal-dependent memory by increasing synaptic and blood vessel density, particularly in the dentate gyrus, a subregion of the hippocampus (8). This flavanol effect on the aging human dentate gyrus has been indirectly confirmed via neuroimaging approaches sensitive to synaptic and blood vessel density (6). The dentate gyrus is distinguished by the fact that it supports neurogenesis after birth (17, 18), a feature that has been hypothesized to “imprint” upon the cells of the dentate gyrus a distinct molecular expression profile (2). Interestingly, across the cortex, the dentate gyrus appears to differentially express BDNF (brain-derived neurotrophic factor) and VEGF (vascular endothelium growth factor), and previous studies showed that flavanols can up-regulate BDNF and VEGF (19, 20). The properties of the neuropsychological instruments provide additional insights into the implicated anatomical biology. While no neuropsychological test evaluates a neuroanatomical region with perfect specificity and sensitivity, the ModRey task was designed to assess hippocampal function (21), which has been validated with neuroimaging (22) and shows age-related decline (6). Similarly, the Flanker test taps prefrontal cortex function and evidences age-associated decline (16). In this study, the flavanol intervention selectively improved ModRey test performance. While we cannot rule out the possibility that other neurobiological systems or differential psychometric properties of the outcomes influenced test performance over time, together with the growing literature on the mechanisms underlying the impact of flavanols on cognition, the findings implicate the hippocampus primarily. Whether and precisely how these factors interact to link flavanols to hippocampal function needs to be mechanistically tested in future studies.

One limitation of the study is that while there is general concordance between analyses stratified by habitual diet quality (Fig. 2) or by gVLM (Fig. 3), two differences were observed. First, at baseline, the high gVLM tertile group randomized to the flavanol intervention arm had higher ModRey scores than all other groups, including those in the high gVLM tertile group randomized to the placebo arm. Second, while the flavanol intervention in the high gVLM tertile group showed no increase at the 12-mo follow-up when compared to the placebo group, an interaction was observed, suggesting the possibility of a relative memory decline. We believe that the second observed difference relates to the first in that those in the high gVLM tertile at baseline started with higher memory scores, thus potentially reflecting a limitation of randomization. We note that, as illustrated in Fig. 4, those participants in the low tertile show an increase in gVLM levels after 1 y of flavanol intervention, while those in the highest tertile, who at baseline are above the population mean, do not. Although the biological relevance of this apparent “ceiling effect” in the absorption, distribution, metabolism, and excretion of flavanols requires further study, the presence of ceiling effects is a common observation in the context of nutrient intake.

While the effect size of the flavanol-associated increase in memory might be considered small, sustained and small effect sizes are often characteristic of randomized clinical trials that are based on diet and lifestyle modifications in generally healthy people and at a population level are often meaningful to health maintenance, dietary guidance, and public health (23–25). Furthermore, the effect size in COSMOS-Web was potentially constrained by various factors. Participants were stratified into relative tertiles based on the distribution of the sample and not preselected based on absolute levels or a priori–defined deficiencies. Relatedly, among those in the lowest baseline diet or gVLM tertile, flavanol supplementation appeared to restore memory performance by repleting their flavanol levels back to levels associated with better memory function. The degree of improvement was therefore limited by a memory performance-specific ceiling effect imposed by those participants in the higher tertiles, which defines “memory potential” in the face of relatively higher flavanol levels, and by the degree of a relative flavanol deficit at baseline. Thus, future studies that test participants at lower ends of either habitual diet quality scores or the gVLM level are likely to demonstrate larger effects. Future studies will also need to include participants from more diverse backgrounds, with greater representation of the entire population; the predominantly White and highly educated characteristics of the participants included in this study limit the generalizability of our findings.

Compared to common late-life cognitive diseases such as neurodegenerative disorders, the pathophysiology of cognitive aging is distinct and less pernicious, characterized by synaptic dysfunction, not by neuronal cell death (26). Synapses and their function are more likely to be sensitive to relatively subtle environmental factors such as nutritional status. Just like there are specific constituents of our diets that are vital for the developing brain, the current findings together with previous studies suggest that dietary flavanols are among those factors that can contribute to maintaining the health of the aging brain.

We are not only living longer, but we are living cognitively more demanding lives. Our findings suggest that flavanol consumption might be considered in future dietary recommendations, perhaps together with the flavanol biomarker, specifically geared toward preventing or improving brain health in later life.

## Materials and Methods

### Study Design.

#### COSMOS.

COSMOS-Web was an ancillary study to the COSMOS (NCT02422745) (14, 27), which was designed to examine the effects of cocoa extract and multivitamin supplementation on total cardiovascular disease and total invasive cancer; here, we report on the cocoa extract intervention only. COSMOS included men (≥60 y old) and women (≥65 y old) in a 2×2 factorial, randomized, double-blind, placebo-controlled trial. COSMOS recruited participants from three main sources, including the Women’s Health Imitative (WHI), respondents to recruitment efforts for the VITamin D and OmegA-3 TriaL (NCT01169259) who were not ultimately randomized into the trial, and those identified through mass mailings and media efforts. Contact was made with nearly three million prospective participants. There were 21,442 participants ultimately enrolled and randomized into the trial.

The interventions in COSMOS included capsules containing 500 mg/d cocoa flavanol extract [including 80 mg (–)-epicatechin] or a placebo provided by Mars Edge (for full compositional details of the test material, see ref. 28) and a Centrum Silver daily multivitamin or placebo provided by Pfizer Consumer Healthcare (now GSK Consumer Healthcare; for full compositional details of the test material, see ref. 27). The overall COSMOS trial had a median treatment period of 3.6 y. The trial included women aged ≥65 y and men aged ≥60 y. Additional inclusion criteria were willingness to participate in the trial as evidenced by completion of informed consent and baseline forms. Major exclusion criteria were history of myocardial infarction or stroke; diagnosis of invasive cancer other than nonmelanoma skin cancer in the last 2 y prior to enrollment; serious illness that would preclude participation; taking cocoa extract, multivitamins, high-dose vitamin D, or high-dose calcium supplements and not willing to forego use during the trial; extreme sensitivity to caffeine; less than 75% compliance to study procedures during at least a 2-mo run-in period; and inability to communicate in English.

### COSMOS-Web.

The current study was ancillary to COSMOS and termed COSMOS-Web (NCT04582617). COSMOS-Web was motivated by emerging literature over the past decades suggesting that dietary patterns mediate aspects of cognitive aging, that flavanol consumption may be one etiological source of hippocampal-dependent memory decline, and that supplementation with dietary flavanols can improve hippocampal-dependent memory (6, 8, 9). Our overarching prespecified hypotheses were that intervention with flavanol supplementation would improve hippocampal-dependent memory over a 1-y period and that this effect would be more prominent among individuals with poorer baseline diets (9). The COSMOS-Web prespecified primary outcome was performance on the ModRey test (21) following 1 y of intervention. Prespecified secondary analyses included testing effect modification by self-reported baseline dietary quality. We tested the specificity of this effect by contrasting performance changes on hippocampus-dependent memory tests (6) to performance changes on the Flanker test (16), a measure putatively mediated by the prefrontal cortex, and explored outcomes across measures at the 2- and 3-y follow-up visits. Our original primary cognitive outcome measure was performance on the ModBent test, but prior to completion of data collection, we changed it to performance on the ModRey test because of concerns about the psychometric properties of the ModBent (9); this change was made completely independent of any statistical analyses of the current study data and was documented in clinicaltrials.gov. Secondary analyses regarding the multivitamin intervention will be reported separately. COSMOS-Web assessment procedures were conducted remotely over the internet via self-guided online assessment instrumentation. Two major innovations developed during the COSMOS-Web trial included the introduction of an objective biomarker to capture flavanol consumption at baseline and follow-up from previously collected spot urine and the deployment of a fully remote online neuropsychological battery to test efficacy.

### Participant Recruitment.

During the COSMOS enrollment process, all participants who agreed to participate in the parent trial were also mailed an invitation to enroll in the COSMOS-Web study, during the enrollment period of August 2016 to March 2018. In addition to the COSMOS inclusion criteria, the COSMOS-Web inclusion criteria required that prospective participants 1) had access to, and the ability to use, an internet-connected computer, and 2) were willing to complete the COSMOS-Web assessment annually for up to 3 y. Participants who agreed to enroll in the COSMOS-Web study were mailed instructions on how to access the study online. All participants received a $15 gift card for attempting each COSMOS-Web assessment, regardless of completion. COSMOS-Web received ethical approval from the Institutional Review Board at Brigham and Women’s Hospital/Mass General Brigham. The CONSORT diagram is presented in Fig. 1.

### Online Platform.

The COSMOS-Web cognitive battery was self-administered by participants on their desktop or laptop computers. The battery was programmed and administered with the Inquisit-Web platform (Millisecond Software, Seattle, WA). Participants downloaded an Inquisit-Web client and the COSMOS-Web battery on their home computers, where the software was run locally to avoid web-based latency variability for reaction time measurements. Instructions for each task in the battery were shown prior to the task. After completion of the battery, participants’ performance data were automatically uploaded to Inquisit servers and downloaded locally for statistical analysis. A telephone helpline at Brigham and Women’s Hospital was provided to aid participants if they had any technical troubles running the battery.

## Cognitive Outcomes

**Fig. 5.**
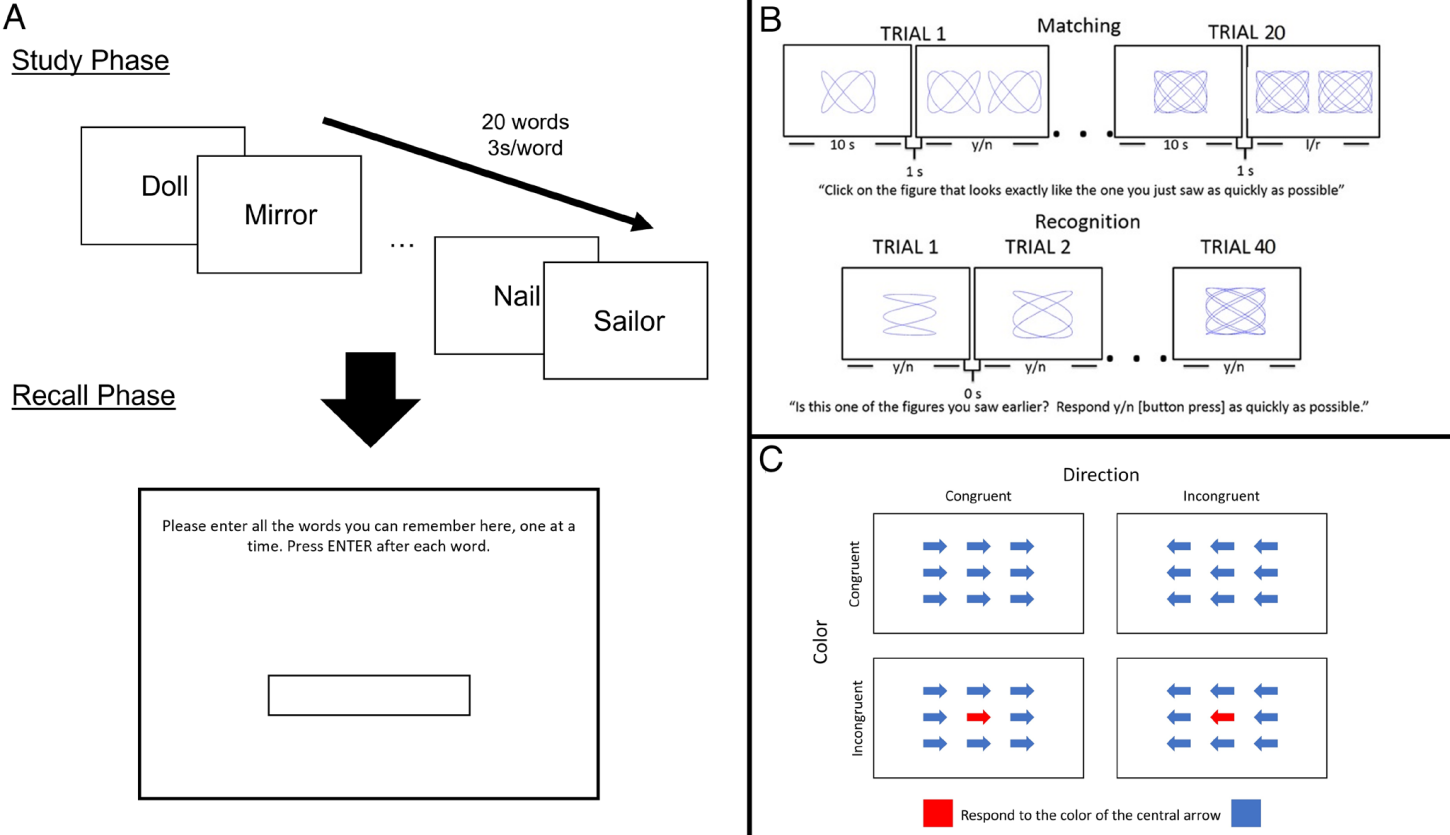
Cognitive outcome measures for COSMOS-Web, including the ModRey (*A*), the ModBent (*B*), and the Flanker test (*C*).

The primary and secondary COSMOS-Web cognitive measures included performance on the following tasks, which were adapted for self-administration over the internet (Fig. 5):

1. ModRey. The ModRey, or Modified Rey Auditory Verbal Learning Test, is a list learning and recall episodic memory test (21). We administered an abbreviated version of this test in which participants were displayed in the center of the computer screen a list of 20 words, one at a time, for 3 s each and instructed to try to retain each word in memory. Recall was assessed immediately after the word list was presented a single time and again after a ~20-min delay (i.e., after the ModBent and Flanker tasks were administered). On the recall trials, participants typed in as many words as they could remember from the learning list. To account for the possibility of typographical errors, responses with at least an 80% match to the letters in the original word were scored as correct, unless they spelled out a different common English word (e.g., for “river”, “riber” would be counted as correct, but “rider” would not). The primary outcome measure used in the trial was the number of words provided on the immediate recall trial. Four counterbalanced versions of this task were used, with independent word lists drawn from the ModRey verbal memory task; we previously demonstrated psychometric similarity among alternate lists (21). One-year change in ModRey immediate recall scores was prespecified as the primary outcome; change in the scores over subsequent years was a secondary outcome.
2. Color/directional flanker test. The Flanker test is a measure of executive control (16). In each trial, participants are asked to respond to the color of a central arrow, surrounded by other arrows. Trials vary on color congruency (whether the surrounding arrows are the same color as the central arrow) and directional congruency (whether the direction of the arrow matches the correct response). The outcome measure used in analyses was the size of the directional flanker effect, which is the difference in reaction time (RT) between directionally congruent and incongruent trials. Changes in Flanker test scores from baseline to years 2 and 3 were secondary outcomes.
3. ModBent. The ModBent, or Modified Benton Visual Retention Test, is a test of mnemonic pattern separation (6). We administered a shortened version of the test adapted for use over the internet. Participants incidentally studied geometric stimuli during a matching task. Subsequently, they completed a forced-choice recognition task on those stimuli. The primary outcome measure is the RT to the correctly rejected lures during the recognition trials (8). The short-form version presented in the COSMOS-Web study is an abbreviated version with half the number of trials of the original ModBent task. Changes in ModBent scores from baseline to years 2 and 3 were secondary outcomes.

### Statistical Analysis.

Demographic, clinical, and baseline cognitive data were summarized by the randomized treatment group, and differences were tested with *t* tests and chi-square tests. Based on recommendation not to focus on statistical tests of baseline differences, we also calculated standardized absolute mean differences. Values >0.25 were considered to be nontrivial imbalance due to chance. Differences were examined within the intent-to-treat sample and also within the subset who had baseline gVLM measured. We performed all analyses on the intent-to-treat (29) sample, which included all randomized participants who had at least one follow-up measurement completed (year 1, 2, or 3) regardless of pill compliance. The prespecified primary end point was at year 1.

The effect of the randomized dietary flavanol intervention on the change in the primary outcome (ModRey: Immediate Recall from baseline to 1 y) and the secondary outcomes (ModRey: Immediate Recall from baseline to 2 and 3 y; ModBent: Correct Rejection and Flanker: Direction Effect from baseline to 1, 2, and 3 y) was tested with linear mixed-effects models with no additional control for multiple testing. Predictors included baseline measures of the respective outcome, dichotomous treatment (dietary flavanol or placebo), categorical year, treatment × year interaction, and a random intercept to account for repeated measures over time. Missing data for follow-up were accounted for within the mixed-effects model under a missing at random assumption. Cohen’s d effect sizes were calculated for all treatment effects to allow a direct comparison of magnitude using the baseline SD of each variable across both groups.

The associations between baseline cognitive measures (ModRey: Immediate Recall; ModBent: Correct Rejection; and Flanker: Direction Effect) and baseline aHEI and gVLM were examined with Pearson correlation coefficients. Linear regression with baseline cognitive measures as the outcome predicted by the aHEI or gVLM (continuous and also categorized into tertiles) further tested the association unadjusted and adjusted for sex, age, education, and race.

Additional secondary analyses tested for treatment effect modification on the cognitive measures across time by baseline healthy eating and gVLM. Note that effect modification by gVLM levels was not originally a prespecified analysis because at the time of study registration, this biomarker had not been accredited. However, we included it in our investigations after it became available, as it complemented our existing approaches to investigate a potential link between habitual diet quality, flavanol consumption, and cognitive aging in a more direct, specific, and objective way. Specifically, tertiles of the baseline values of the aHEI or the gVLM were included (separately) in the mixed-effects model along with their interaction with the dichotomous treatment, time, and time by treatment. The test of interaction between the baseline aHEI and treatment or baseline gVLM and treatment was used to determine whether any treatment effects were different by baseline tertile. When significant interactions were found, post hoc tests of the treatment effect within each tertile of the aHEI and gVLM were performed. Moreover, a treatment effect contrast within each tertile of the aHEI and gVLM averaging the specific effects over all three follow-up time points was also obtained. These contrasts and tests were obtained from the same overall mixed-effects model with interactions. Per protocol analyses were also performed restricting to the subset of subjects who met the definition for pill compliance, having ≥75% compliance taken at year 1. This subset of year 1 pill compliers was used for all PP analyses, even those including year 2 and 3 outcomes to ensure consistency of the study sample being analyzed. Of note, year 2 and 3 pill compliance among year 1 pill compliers was very high, 93.5% in year 2 and 90.6% in year 3. Analyses were carried out using SAS 9.4.

## Supplementary Material

Appendix 01 (PDF)

## Data Availability

All data and code used in the analyses will be made available through Dryad at https://doi.org/10.5061/dryad.m905qfv69 (30).
