# Supplementary material for: Dietary flavanols restore hippocampal-dependent memory in older adults with lower diet quality and lower habitual flavanol consumption
Source: Proc Natl Acad Sci U S A. 2023 May 30;120(23):e2216932120. doi: 10.1073/pnas.2216932120 (PMC10265949; doi:10.1073/pnas.2216932120)

Dietary flavanols restore hippocampal-dependent memory in older adults with lower diet quality and habitual flavanol consumption

Adam M. Brickman[1,2,3,*], Lok-Kin Yeung[1,2,3], Daniel M. Alschuler[4], Javier I. Ottaviani[5], Gunter G.C. Kuhnle[6], Richard P. Sloan[4,7], Heike Luttmann-Gibson[8,9], Trisha Copeland[8], Hagen Schroeter[5], Howard D. Sesso[8,10], JoAnn E. Manson[8,10], Melanie Wall[4,7], Scott A. Small[1,2,3,7,*]

1. Taub Institute for Research on Alzheimer's Disease and the Aging Brain, Vagelos College of Physicians and Surgeons, Columbia University, New York, NY
2. Gertrude H. Sergievsky Center, Vagelos College of Physicians and Surgeons, Columbia University, New York, NY
3. Department of Neurology, Vagelos College of Physicians and Surgeons, Columbia University, New York, NY
4. New York State Psychiatric Institute, New York, NY
5. Mars Edge, USA, McLean, VA
6. Hugh Sinclair Unit of Human Nutrition, Department of Food and Nutritional Sciences, University of Reading, Reading, UK
7. Department of Psychiatry, Columbia University Irving Medical Center, New York, NY
8. Division of Preventive Medicine, Department of Medicine, Brigham and Women's Hospital, Harvard Medical School, Boston, MA
9. Department of Environmental Health, Harvard T.H. Chan School of Public Health, Boston, MA
10. Department of Epidemiology, Harvard T.H. Chan School of Public Health, Boston, MA

[*]Corresponding authors
Adam M. Brickman
Taub Institute for Research on Alzheimer's Disease and the Aging Brain
Vagelos College of Physicians and Surgeons
Columbia University
630 West 168th Street
New York, NY 10032 USA
Tel: +1 212 342 1348
Email: amb2139@columbia.edu

Scott A. Small
Taub Institute for Research on Alzheimer's Disease and the Aging Brain
Vagelos College of Physicians and Surgeons
Columbia University
630 West 168th Street
New York, NY 10032 USA
Tel: +1 212 305 1269
Email: sas68@columbia.edu

Classification: Biological Sciences, neuroscience
Keywords: cognitive aging, hippocampus, dietary flavanols

Supporting Information

Supplemental tables.

Supplemental Table 1. Baseline demographic, dietary, and cognitive testing by randomized assignment among 1361 COSMOS-Web participants with gVLM measurements

	Total Sample (N=1361)		Placebo (N=662)		Dietary Flavanol (N=699)			
**Measure**	**N**	**% or M (SD)**	**N**	**% or M (SD)**	**N**	**% or M (SD)**	**Absolute Standardized Mean Difference[a]**	**p-value[b]**
**Demographics**								
Age	1361	70.6 (4.4)	662	70.7 (4.4)	699	70.6 (4.3)	0.024	0.659
Gender								0.780
Male	515	37.8%	253	38.2%	262	37.5%	0.015	
Female	846	62.2%	409	61.8%	437	62.5%	0.015	
Race								0.270
White	1297	95.3%	630	95.2%	667	95.4%	0.012	
African American	24	1.8%	15	2.3%	9	1.3%	0.074	
Hispanic	17	1.2%	6	0.9%	11	1.6%	0.060	
Asian or Pacific Islander	12	0.9%	4	0.6%	8	1.1%	0.058	
Other or Unknown	11	0.8%	7	1.1%	4	0.6%	0.054	
Education								0.805
Missing	12	0.9%	5	0.8%	7	1.0%	0.026	
High school	60	4.4%	26	3.9%	34	4.9%	0.046	
College	557	40.9%	274	41.4%	283	40.5%	0.018	
Post-college	732	53.8%	357	53.9%	375	53.6%	0.006	
**Baseline dietary**								
AHEI	1296	43.4 (10.5)	631	43.6 (10.5)	665	43.1 (10.6)	0.044	0.429
Urine gVLM	1361	9.5 (14.7)	662	9.4 (15.5)	699	9.6 (14.0)	0.008	0.878
**Baseline testing**								
ModRey immedate recall	1361	7.09 (3.23)	662	6.97 (3.14)	699	7.21 (3.31)	0.074	0.170
ModBent correct rejection	1359	2755.43 (1425.52)	662	2800.02 (1574.56)	697	2713.08 (1267.56)	0.061	0.261
Flanker direction effect	1360	33.04 (55.46)	661	33.28 (56.25)	699	32.81 (54.74)	0.008	0.876

[a]Standardized absolute mean difference is calculated as the difference between treatment groups divided by the overall standard deviation. Values >0.25 are considered to be non-trivial imbalance due to chance.

[b]p-values for t-tests of continuous variables and chi-square tests of categorical variables

Supplemental Table 2. Longitudinal mixed effect model results - Primary (Modrey Immediate Recall) and Secondary (Modrey Retention, Flanker Direction) outcomes by randomized treatment - pill compliers

	Placebo				Dietary flavanol				Treatment Difference		
Timepoint	N	Raw mean (SD)	Raw mean change from baseline (SD)	Within group p-Value	N	Raw mean (SD)	Raw Mean change from baseline (SD)	Within group p-Value	Mean (SE)[a]	Cohen's d	p-value
ModRey: Immediate Recall											
Baseline	1671	7.16 (3.25)			1598	7.18 (3.20)					
Year 1	1602	7.66 (3.41)	0.50 (3.34)	<.001	1536	7.83 (3.26)	0.63 (3.31)	<.001	0.14 (0.10)	0.043	0.184
Year 2	1471	7.98 (3.37)	0.78 (3.39)	<.001	1411	8.11 (3.25)	0.86 (3.40)	<.001	0.09 (0.11)	0.027	0.419
Year 3	1392	8.19 (3.40)	1.02 (3.29)	<.001	1351	8.25 (3.39)	0.99 (3.32)	<.001	0.01 (0.11)	0.002	0.949
ModBent: Correct Rejection											
Baseline	1635	2745.44 (1349.43)			1572	2796.46 (1368.78)					
Year 1	1535	2776.25 (1284.70)	29.88 (1517.92)	0.648	1469	2863.27 (1472.95)	65.48 (1713.55)	0.011	71.04 (47.33)	0.052	0.133
Year 2	1413	2772.55 (1334.81)	25.58 (1551.05)	0.697	1361	2821.95 (1365.53)	35.27 (1635.66)	0.188	32.73 (49.06)	0.024	0.505
Year 3	1339	2815.93 (1313.60)	74.64 (1513.76)	0.144	1294	2813.52 (1434.56)	42.25 (1618.85)	0.242	-9.48 (50.19)	0.007	0.850
Flanker: Direction Effect											
Baseline	1637	31.36 (57.38)			1577	30.82 (57.31)					
Year 1	1538	26.53 (58.10)	-5.14 (74.54)	<.001	1473	27.80 (49.50)	-2.75 (69.81)	0.017	1.45 (1.92)	0.025	0.451
Year 2	1409	26.96 (49.58)	-4.51 (70.10)	0.002	1365	27.22 (50.89)	-4.06 (69.17)	0.006	0.41 (2.00)	0.007	0.836
Year 3	1335	23.07 (54.84)	-8.12 (76.34)	<.001	1302	24.06 (54.96)	-7.36 (73.94)	<.001	0.97 (2.05)	0.017	0.635

[a]Treatment effect controlling for baseline.

Supplemental Table 3. Overlap between tertiles defined by baseline gVLM levels and tertiles defined by baseline aHEI levels (with cell percentages). Table does not include missing data.

	Low aHEI tertile	Middle aHEI tertile	High aHEI tertile	Total
Low gVLM tertile	148 (11.4%)	137 (10.6%)	141 (10.9%)	426 (32.9%)
Middle gVLM tertile	139 (10.7%)	153 (11.8%)	144 (11.1%)	436 (33.6%)
High gVLM tertile	127 (9.8%)	165 (12.7%)	142 (11.0%)	434 (33.5%)
Total	414 (31.9%)	455 (35.1%)	427 (32.9%)	1296 (100.0%)

Supplemental figures.

Supplemental Figure 1. The association of baseline gVLM concentrations as a continuous variable (color coded by tertile) with change in ModRey scores at Years 1, 2, and 3 in the placebo group (upper row) and dietary flavanol intervention group (lower row). Fit line represents locally estimated scatterplot smoothing (LOESS).

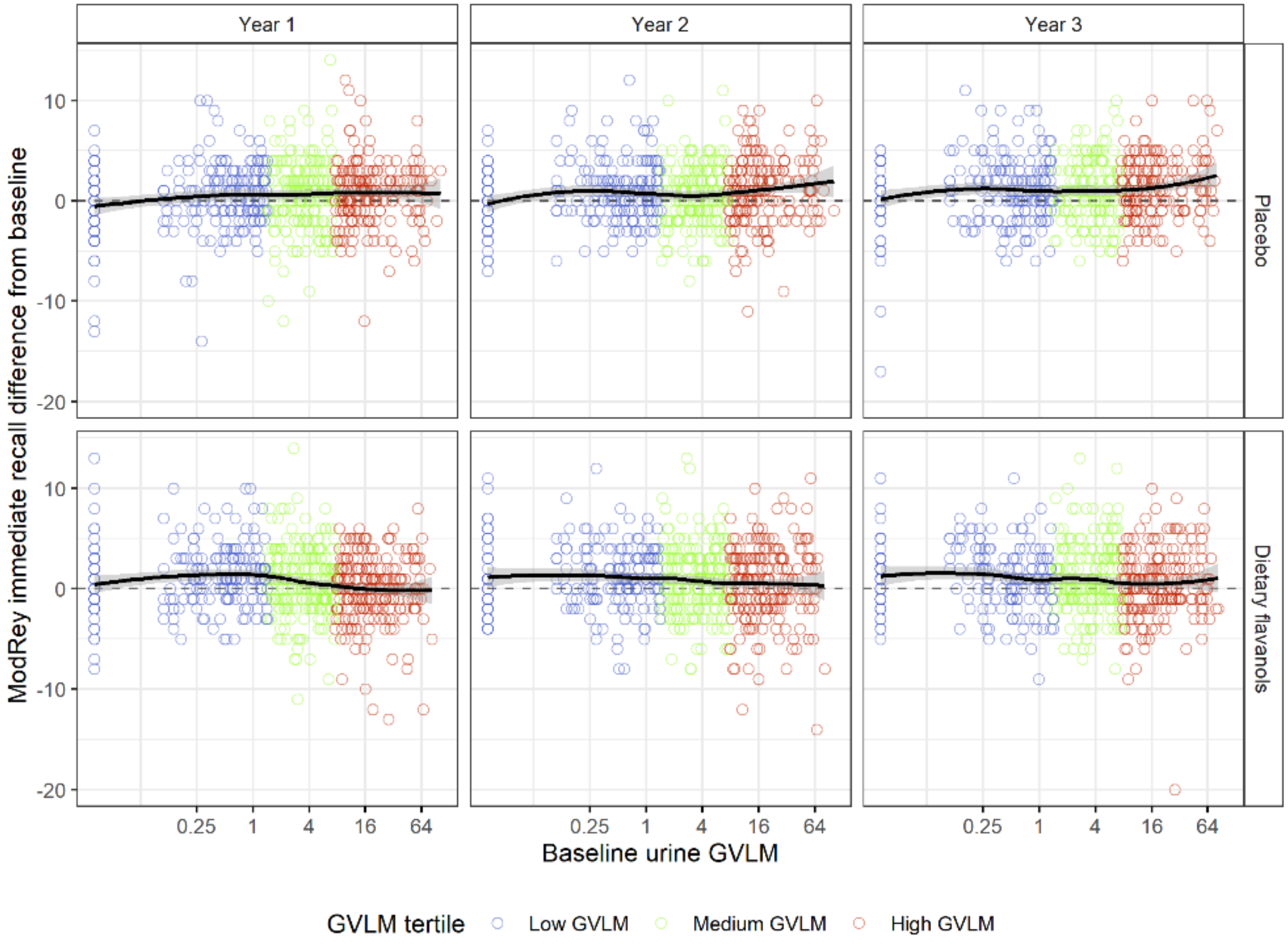

Supplemental Figure 2. Relationship between change in gVLM levels and change in ModRey immediate memory scores.

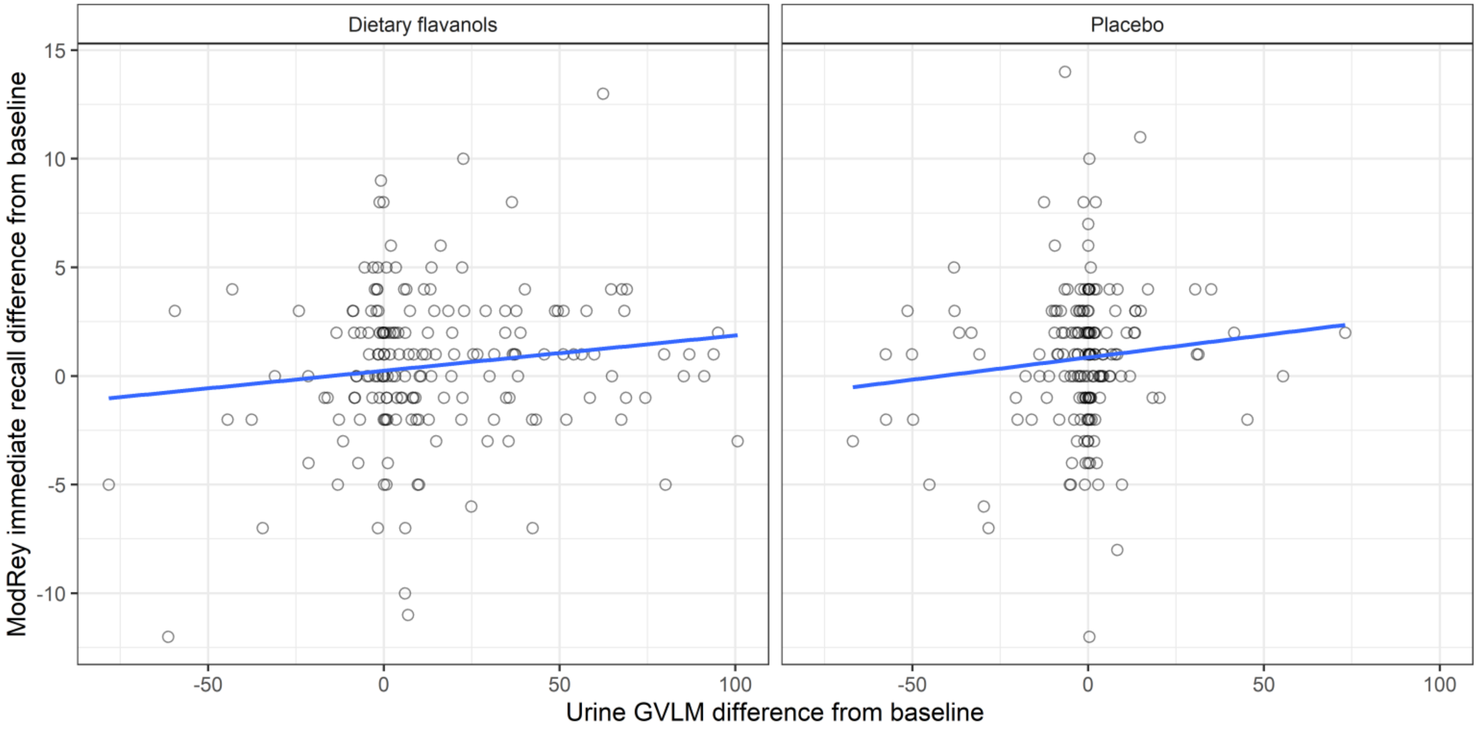

Supplement: Supplementary file 1 — Appendix 01 (PDF) [file pnas.2216932120.sapp.pdf]
